# Quantitative assessment of perfusion-weighted magnetic resonance imaging for the differential diagnosis of various intraocular lesions

**DOI:** 10.1007/s00234-026-04069-7

**Published:** 2026-06-16

**Authors:** Iris Mulder, Khanh Vu, Teresa Ferreira, Lisa Klaassen, Jan-Willem Beenakker

**Affiliations:** 1 Department of Ophthalmology, Leiden University Medical Center, Leiden, Netherlands; 2 Department of Radiology, Leiden University Medical Center, Leiden, Netherlands; 3 HollandPTC, Delft, Netherlands

**Keywords:** Magnetic resonance imaging, Ocular oncology, Uveal melanoma, Perfusion, Fluorescein angiography, Tofts model

## Abstract

**Purpose:**

To conduct an exploratory analysis of quantitative and semi-quantitative perfusion-weighted MRI (PWI) parameters across various intraocular lesions and assess their relationship with fluorescein angiography (FAG) features.

**Methods:**

Thirty-eight patients with twelve different diagnoses who underwent 3T dynamic contrast-enhanced MRI (DCE-MRI) of the eye were included, with diagnosis confirmed either by histopathology or clinical course of the disease. Tofts pharmacokinetic modelling was used to calculate K^trans^, v_e_, and k_ep_ values. Semi-quantitative relative peak intensity and outflow percentage were derived from time-intensity curves and compared with quantitative parameters. FAG images were evaluated for blocking effects, pinpoint and diffuse leakages.

**Results:**

Median values of quantitative and semi-quantitative parameters varied across lesion types. Haemangiomas showed the highest K^trans^ (median = 6.35 min^− 1^) and k_ep_ (median = 3.8 min^− 1^). Uveal melanomas showed high K^trans^ (median = 0.68 min^− 1^) and k_ep_ (median = 2.45 min^− 1^) in comparison with choroidal naevi and metastases. Quantitative and semi-quantitative parameters showed significant correlation across all intensity groups (all R^2^ > 0.36, all *p* < 0.04). The correlation between K^trans^ and relative peak intensity differed for hypo- and isointense lesions versus hyperintense lesions (both *p* < 0.002). Statistical comparison between individual diagnoses was not possible due to limited sample size per diagnosis. Quantitative PWI parameters showed correspondence with FAG features.

**Conclusion:**

This study provided quantitative and semi-quantitative PWI parameters for various intraocular lesions. Semi-quantitative PWI parameters may provide comparable information on lesion vascularity when stratified by T1 signal intensity.

**Supplementary Information:**

The online version contains supplementary material available at https://doi.org/10.1007/s00234-026-04069-7.

## Introduction

 In ocular oncology, differentiating uveal melanomas (UM) from other intraocular lesions can be challenging. To aid the process of differential diagnosis, various imaging techniques are being used, including fundus photography, optical coherence tomography (OCT), ultrasonography, indocyanine green angiography (ICG), and fluorescein angiography (FAG) [1]. However, conventional ophthalmic imaging methods are not always conclusive in differentiating intraocular lesions, particularly in cases complicated by intraocular hemorrhage or peripherally located lesions. In such cases, intraocular lesions can be visualized and characterized by magnetic resonance imaging (MRI). MRI is increasingly recognized for its ability to provide both anatomical and functional information, particularly in retinoblastomas [2–5] and UM [6–8].

As part of the ophthalmic diagnostic evaluation, intraocular lesions can be assessed optically based on their vascularization characteristics [9]. To characterize these vascularization patterns, FAG is a commonly used imaging technique in which a fluorescent dye is injected intravenously, followed by sequential optical imaging to evaluate the choroidal and retinal circulation. Differences in vascular patterns have been reported for various intraocular lesions [10]. However, FAG only visualizes the retinal surface of the lesion, whereas perfusion weighted MRI (PWI) assesses the entire volume. A commonly used approach for PWI is dynamic contrast-enhanced (DCE)-MRI, which involves the administration of a gadolinium-based contrast agent while acquiring T1-weighted images every few seconds over several minutes [11–13]. The resulting data can be evaluated semi-quantitatively using relative signal intensity, or quantitatively using pharmacokinetic modelling (PKM). A widely used method for PKM is the Tofts model [14], which describes the transfer of gadolinium from the blood to extracellular space by several key parameters. The parameter K^trans^ represents the rate at which gadolinium moves from the blood plasma into the tissue, affected by both blood flow and vascular permeability, while v_e_ indicates the proportion of tissue volume that is occupied by extracellular extravascular space. The ratio of K^trans^ and v_e_ is k_ep_, and corresponds to the rate at which gadolinium returns from extravascular space to blood plasma.

Although PWI has been widely used in the assessment of orbital tumours [15–17], its application to intraocular lesions is mostly limited to UM [18–20]. Other intraocular lesions include malignant lesions such as metastases, adenoma, and lymphomas, as well as benign lesions such as naevi, haemangiomas, schwannomas, leiomyomas, melanocytomas, and peripheral exudative hemorrhagic chorioretinopathy (PEHCR). The ophthalmic presentation of these lesions may resemble UM [21], leading to diagnostic uncertainty and, in some cases, the need for an invasive biopsy [22]. Additionally, in a subset of patients, ophthalmic imaging is limited by factors such as dense cataract, vitreous hemorrhage, or lesions located directly behind the iris [7]. As treatment strategies differ between lesion types, histological confirmation was historically required for definitive diagnosis in such cases [22]. Over the past decade, however, MRI has increasingly been used as a complementary imaging modality [6, 7]. Since anatomical imaging alone is often limited to assessing the lesion’s pigmentation [6], quantitative MRI techniques such as diffusion‑ and perfusion‑weighted imaging are commonly employed to further differentiate between lesion types [7]. DCE-MRI can directly impact patient management by reducing the need for invasive intraocular diagnostic biopsies [22]. In addition, DCE-MRI provides early assessment of radiotherapy treatment before standard ophthalmic imaging, which can be inconclusive due to pseudo-progression [18, 20].

Furthermore, because UM is often pigmented, PKM is reportedly more accurate for intraocular lesions [20] compared to the clinically used semi-quantitative methods. Given the varying degree of pigmentation across different types of intraocular lesions [23], it remains unclear how PWI should be incorporated in the differential diagnosis. Furthermore, it is uncertain whether quantitative PKM analysis is necessary or if commonly used semi-quantitative metrics are sufficient. Therefore, the aim of this study is to describe both the quantitative and semi-quantitative PWI parameters in a cohort of various intraocular lesions and to explore the extent to which quantitative PWI provides additional diagnostic information over a semi-quantitative analysis. In addition, as both FAG and PWI offer information on perfusion characteristics, this study aims to evaluate the correspondence between quantitative PWI parameters and the ophthalmic assessment of FAG images.

## Methods and materials

### Patient population

All patients who received a 3T MRI of the eye with a surface coil between April 2020 and February 2024, which included DCE-MRI scans, were considered for inclusion. Additional inclusion criteria were a definitive diagnosis of an intraocular lesion other than UM, confirmed either by histopathology or by the clinical course of the disease. An additional 11 UM patients were included for comparison, some of which were previously analyzed by Klaassen et al. [20]. Patients were included retrospectively, based on clinical scans originally performed for differential diagnosis, or prospectively, with MRI acquisition after the diagnosis was made. For retrospectively included patients, the requirement for informed consent was waived by the local ethics committee as all procedures were performed as part of clinical care. For prospectively included patients, written informed consent was obtained prior to participation. Ethical approval for this part of the study was granted by the local ethics committee (protocol number: 23-2070) and adhered to the principles of the Declaration of Helsinki.

### Image acquisition and processing

Patients were scanned using a 3T MRI scanner (Ingenia Elition, Philips Healthcare, Best, the Netherlands) equipped with a 4.7 cm surface receive coil (Philips Healthcare), following the protocol described by Ferreira et al. [24]. The MR protocol, detailed in Table 1, included fat-suppressed contrast-enhanced 3D T1-weighted scans, a flip angle (FA) series for T1 mapping, a Dual Refocusing Echo Acquisition Mode (DREAM) sequence for B1+ mapping [25, 26], and a DCE-MRI with a temporal resolution of 1.9 s per dynamic acquisition using the 4D time-resolved MR angiography with keyhole (4D-TRAK) method [27, 28]. The contrast-enhanced 3D T1-weighted scan was acquired with a spatial resolution of 0.8 mm isotropic, and the dynamic time series with a spatial resolution of 1.25 × 1.25 × 1.5 mm^3^ [13]. After the first 6 s of the dynamic series, a bolus of 0.1 mmol/kg gadoterate meglumine (Dotarem; Guerbet, Roissy CdG Cedex, France or Clariscan; GE Healthcare, IL, USA) was administered using a power injector at a rate of 2 mL/second [20].

**Table 1:** Scan parameters [20]. DCE-MRI: dynamic-contrast enhanced magnetic resonance imaging, DREAM: dual refocusing echo acquisition mode [25], SPIR: spectral presaturation with inversion recovery, TE: echo time, TR: repetition time, 4D-TRAK: 4D time-resolved MR angiography with keyhole [27, 28]

	T1-mapping	B1+-mapping	DCE-MRI	3D T1
Acquisition voxel size [mm^3^]	1.3 x 1.5 x 1.5	2.00 x 2.00 x 2.00	1.3 x 1.5 x 1.5	0.80 x 0.80 x 0.80
Reconstruction voxel size [mm^3^]	1.0 × 1.0 × 1.0	1.0 × 1.0 × 2.0	1.0 × 1.0 × 1.0	0.4 × 0.4 × 0.4
TE/TE2/TR [ms]	3.1/-/7.0	2.3/3.6/7.1	2.3/-/4.5	31/-/400
Flip angle [degrees]	2/5/9/15	10	13	90
Fat suppression	Proset 11	SPIR	Proset 11	SPIR
Scan time [mm:ss]	4 × 00:07	00:14	03:59	02:07
Remarks		DREAM sequence	Temporal resolution 1.9s / dynamic, 4D-TRAK	Contrast-enhanced

The FA series, B1+ maps and all timepoints of the DCE-MRI were rigidly registered to the contrast-enhanced 3D T1-weighted scan using Elastix version 4.9.0 [29]. Subsequently, the registered images were rigidly re-registered using an eye mask to correct for eye motion, as proposed by Jaarsma-Coes et al. [13]. Lesion delineation was performed on the contrast-enhanced 3D T1-weighted images using MeVisLab (MeVis Medical Solutions, Bremen, Germany), with a subdivision surface fitting technique [20], followed by manual corrections by an ophthalmic MRI expert. A previously published method [30] was used to automatically determine the lesion’s largest basal diameter and prominence, excluding the sclera, based on the delineations.

### Perfusion analysis

The B1+ data were used to determine the actual applied FA of the FA series and the dynamic scan. To minimize partial volume effects and to account for interobserver variations of approximately 0.4 mm on MRI for UM [31], lesion delineations were eroded. Since the dynamic scan had larger reconstruction voxels than the contrast-enhanced 3D T1-weighted scan, lesions with a prominence larger than 3 mm were eroded using a spherical structuring element with a radius of three reconstruction voxels of the contrast-enhanced 3D T1, while smaller lesions were eroded using a spherical structuring element with a radius of one reconstruction voxel to ensure a sufficient number of voxels remained. Subsequently, PKM based on the simplified Tofts model [14] with a population-based arterial input function (AIF) was performed in Matlab R2024B (MathWorks, Natick, MA, USA) as described by Jaarsma-Coes et al. [13] and Klaassen et al. [20], for the first 90 timepoints of the dynamic series. This analysis resulted in voxel-wise estimates of the parameters K^trans^, v_e_, and k_ep_ for each lesion, with k_ep_ defined as the ratio between K^trans^ and v_e_. The quality of the PKM fits were evaluated visually based on their correspondence with the observed concentration curves.

**Fig. 1:**
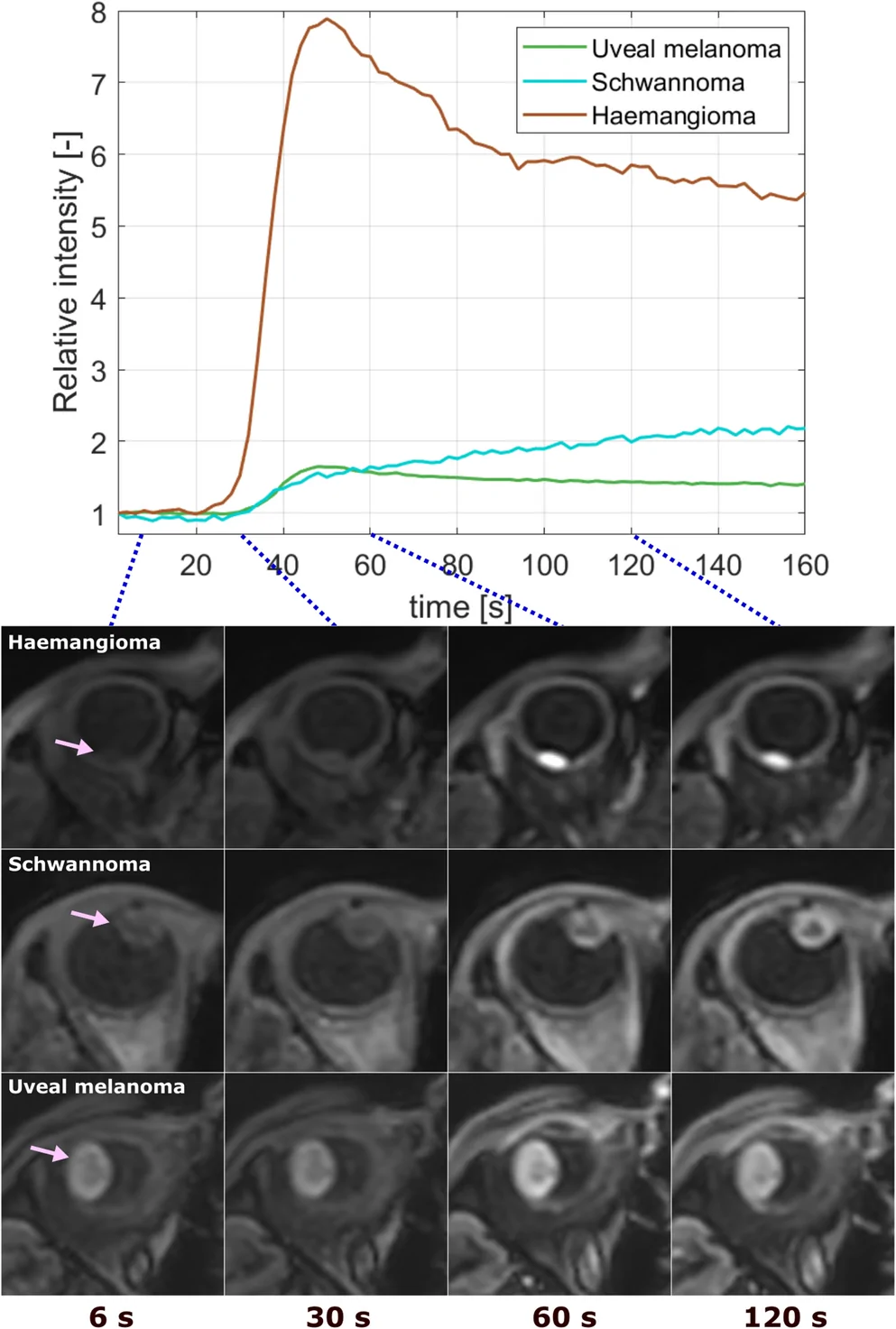
Exemplary TIC of a haemangioma (patient 03), schwannoma (patient 26), and uveal melanoma (patient 28), along with the DCE-MRI at 6, 30, 60 and 120 seconds after the bolus arrival time. More information on individual patients can be found in Supplementary Tables 1 and 2

In addition, commonly used semi-quantitative PWI parameters were manually derived from time-intensity curves (TIC), including relative peak intensity and outflow percentage [32]. Relative peak intensity was defined as the ratio of the peak intensity to the mean signal intensity from the third to fifth timepoints and the outflow percentage was determined at 2 minutes after the peak [32]. TIC were classified as progressive, plateau or wash-out curve types by two independent observers [18, 20]. Any disagreements were discussed to reach consensus. Figure 1 shows the TIC for a haemangioma, schwannoma, and uveal melanoma, along with the DCE-MR-images at 6, 30, 60 and 120 seconds. To evaluate lesion pigmentation, the signal intensity of the lesion on T1-weighted scans were categorized as hyperintense, isointense, or hypointense compared to the choroid, as it was shown that there is a significant relationship between signal intensity on T1 and pigmentation on histopathology for UM [32].

**Fig. 2:**
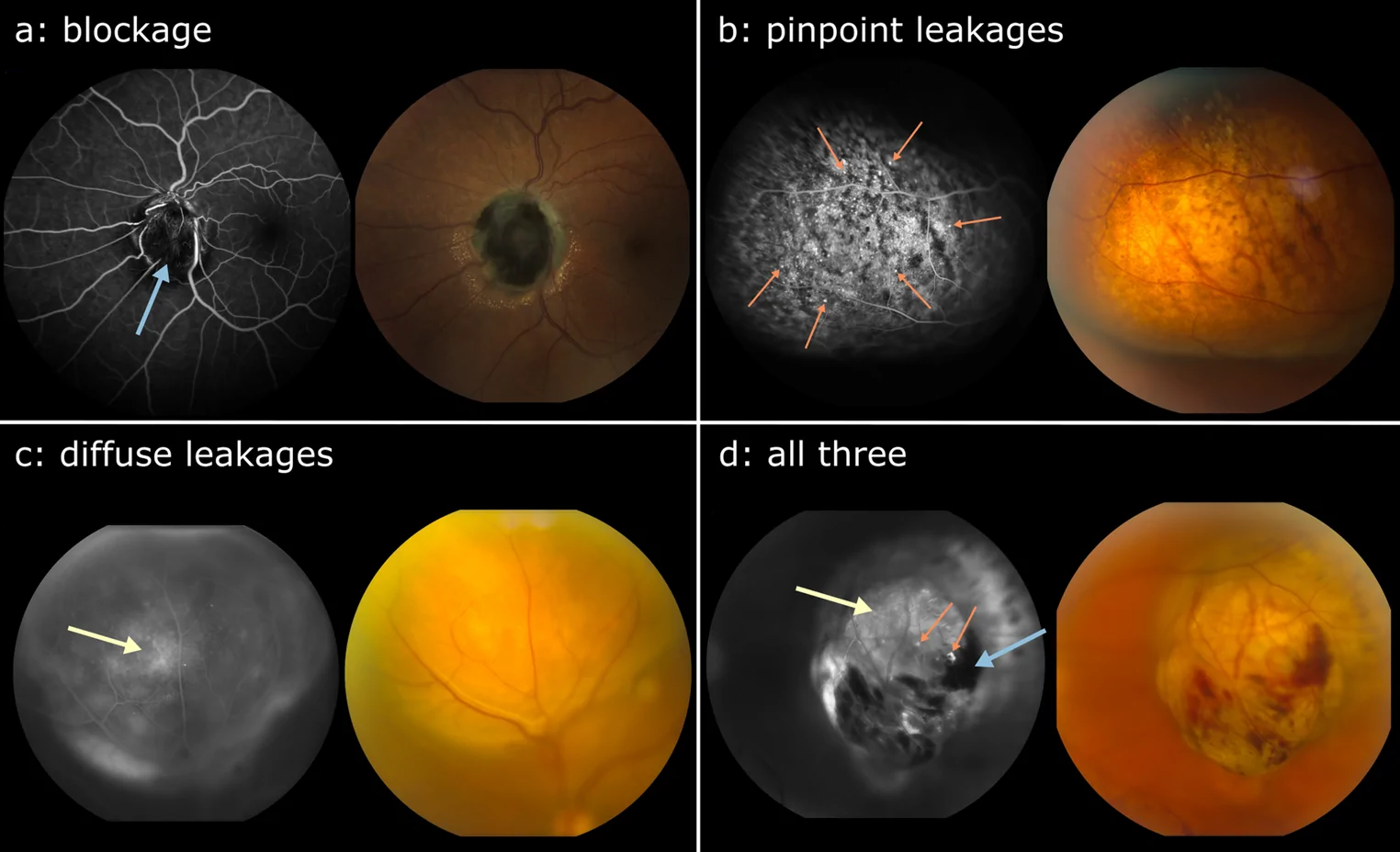
**a** Exemplary FAG and fundus photography of a melanocytoma, with the FAG showing complete blockage of fluorescein signal within the tumour (blue arrow). **b** Exemplary FAG and fundus photography of uveal melanoma, with FAG showing pinpoint leakages (orange arrows) throughout the tumour and blockage at the site of secondary hyperpigmentation. **c** Exemplary FAG and fundus photography of multiple myeloma, with FAG showing diffuse leakage (yellow arrow). **d** Exemplary FAG and fundus photography of uveal melanoma, with FAG showing blockage of fluorescein signal (blue arrow) at the site of hemorrhages, some pinpoint leakages (orange arrows) and diffuse leakage (yellow arrow)

To assess vascularization on ophthalmic imaging, FAG images were assessed when those had been acquired as part of standard clinical care. FAG images were acquired using the Spectralis HRA + OCT system (Heidelberg Engineering, Heidelberg, Germany) and Topcon TRC-50DX (Topcon KK, Tokyo, Japan), after an intravenous injection of 5 mL fluorescein disodium 100 mg/mL (Fresenius Kabi, Huis ter Heide, the Netherlands). Retinal images were captured continuously (approximately 40 images) during the first 80 seconds, followed by one image every 2 minutes until at least 7 minutes after injection. FAG images were evaluated for the presence of pinpoint leakages, diffuse leakage, and blockage of fluorescein signal. Examples of these features on FAG are shown in Fig. 2, along with the corresponding fundus photographs. All FAG images were assessed by an ocular oncologist.

### Statistics

For each lesion, the median and interquartile range (IQR) (25th and 75th percentiles) were determined for the T1, K^trans^, v_e_, k_ep_, relative peak intensity, and outflow percentage. The median and range of the median values per lesion are reported for each diagnosis. Interobserver agreement of TIC classification was evaluated using weighted Cohen’s kappa with equal weights. To assess the impact of pigmentation on the relationship between K^trans^ and relative peak intensity, and between k_ep_ and outflow percentage, linear regression analyses, with an interaction term, were performed for each of the three intensity groups. Analyses were restricted to the overlapping value ranges across groups to ensure comparability. A significant interaction term indicated that the studied relation differs between intensity groups and thus depends on pigmentation [13, 32]. Given the exploratory design of the study and the limited number of patients per diagnosis with available FAG images, differences in K^trans^ and k_ep_ between lesions with and without FAG were only assessed descriptively. Due to the limited number of patients per lesion type, statistical comparisons between lesion types were not performed. No correction for multiple testing was applied, given the exploratory design of the study.

## Results

### Patient population

Initially, 30 patients were included for analysis, of which three were excluded. In one patient, excessive eye movement led to unsuccessful registration of the dynamic time series, and two patients had a very flat lesion that was too small to delineate on 3D T1-weighted MRI. For the remaining 27 patients, diagnosis was confirmed by histopathology in 12 patients, and by clinical presentation and examination in 15 patients. With the additional 11 UM, the total amount of included patients was 38. A complete list of all studied lesion types is reported in Table 2. All lymphomas were secondary intraocular lymphomas with associated systemic lymphoma localizations. Choroidal metastases were from various primary malignancies, of which histopathology confirmed the primary tumour. Information on the diagnostic process for the individual patient can be found in Supplementary Table 1. Included patients had a median age of 60.0 years (range: 10.8–82.9 years) at time of MR acquisition, with a median lesion prominence of 4.7 mm (range: 0.9–11.4 mm) and a median largest basal diameter of 12.9 mm (range: 3.6–21.9 mm). Of these 38 patients, 20 patients also had FAG images available. Characteristics of the individual patients can be found in Supplementary Table 2.

### Perfusion analysis

For all diagnoses, the median K^trans^, k_ep_, relative peak intensity, outflow percentage and the total number of lesions per TIC type and per T1-weighted intensity are reported (Table 2). The median and IQR of the quantitative and semi-quantitative parameters per lesion are reported in Supplementary Table 2.

**Fig. 3:**
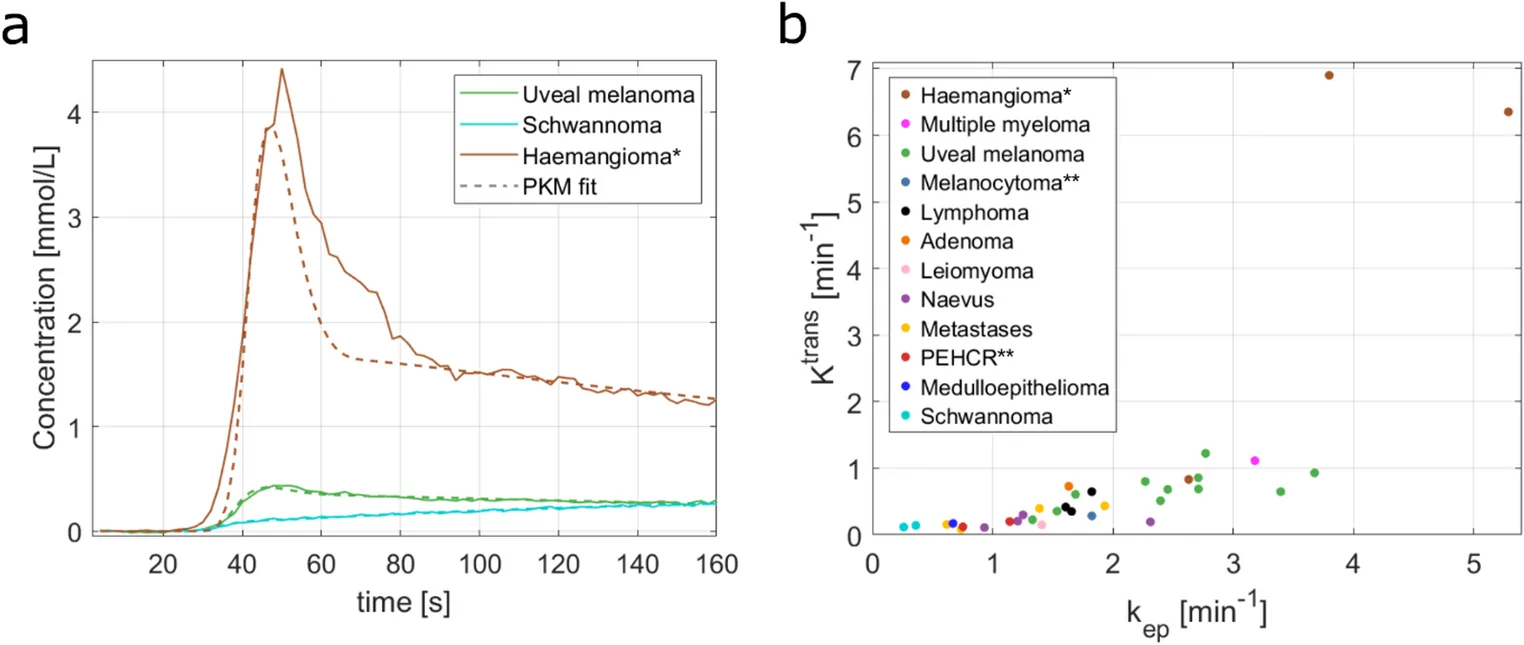
**a** Concentration curves of the same uveal melanoma, schwannoma and haemangioma as depicted in Fig. 1. The dashed line indicates the fit of the Tofts pharmacokinetic model (PKM). The curve of the haemangioma is an example of a failed PKM fit. **b** Median K^trans^ and k_ep_ for all exemplary cases. *quantitative parameters of haemangiomas are exploratory due to poor PKM fit. **Two melanocytomas and two PEHCR had minimal enhancement, resulting in a K^trans^ and v_e_ of (approximately) 0, therefore k_ep_ was not calculated for these lesions and not displayed in the graph

The fits of the PKM sufficiently described the measured concentration data for all lesions that showed enhancement, except for two haemangiomas and two PEHCRs where the PKM fit deviated noticeably from the measured data, as is illustrated in Fig. 3a for one haemangioma. Nonetheless, these two haemangiomas had a considerably higher K^trans^ (6.35 and 6.90 min^− 1^) compared to all other lesions (Fig. 3b), reflecting their highly vascularized nature. Minimal enhancement of the lesion was seen in two of three melanocytomas (K^trans^ < 0.15 min^− 1^), and in two of four PEHCR (K^trans^ < 0.20 min^− 1^). Since K^trans^ and v_e_ of these minimally enhanced lesions was approximately zero, the k_ep_ was considered unreliable for these lesions. The UM showed a relative high K^trans^ (median = 0.68 min^− 1^) and relative high k_ep_ (median = 2.45 min^− 1^) compared to naevi (median K^trans^ = 0.20 min^− 1^, median k_ep_ =1.23 min^− 1^) and metastases (median K^trans^ = 0.28 min^− 1^, median k_ep_ =1.06 min^− 1^). The median values with IQR of K^trans^ and k_ep_ are also shown in Supplementary Fig. 1.

The two independent observers showed a strong agreement in TIC classification, κ = 0.863, *p* < 0.001. All UM, except for one, showed a wash-out type TIC, characterized by a high relative peak intensity (median = 1.67) and a strong negative outflow percentage (median = -33%). The wash-out TIC is commonly associated with malignant lesions and was observed in UM, the multiple myeloma, and the three lymphomas. Benign lesions such as schwannomas and naevi (Table 2) showed progressive TICs. However, not all benign lesions followed this pattern, as the benign adenoma and two haemangiomas demonstrated wash-out TICs as well.

**Fig. 4:**
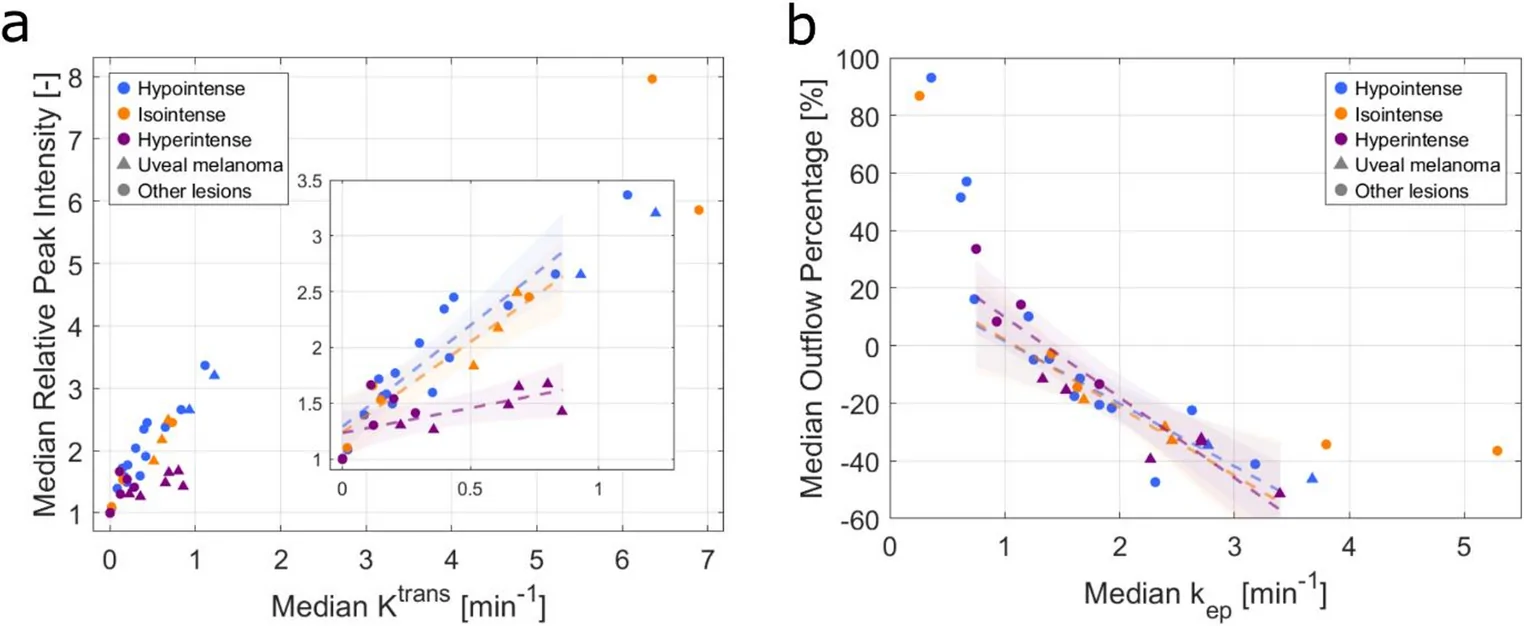
Quantitative and semi-quantitative PWI parameters of exemplary cases of hypo-, iso-, and hyperintense lesions. Signal intensity was evaluated on T1-weighted MR scans. Triangles indicate uveal melanomas, and circles indicate all other kinds of intraocular lesions. **a** Comparison of median relative peak intensity with median K^trans^. **b** Comparison of median outflow percentage with median k_ep_

Six linear regression models for the hypo-, iso-, and hyperintense lesions were tested. K^trans^ showed a significant linear relationship with relative peak intensity, see Fig. 4a (all R^2^ > 0.36, all *p* < 0.04), which was similar between hypo and isointense lesions (*p* = 0.61), but both differed from the hyperintense lesions (both *p* < 0.002). k_ep_ showed a significant linear relationship with outflow percentage, see Fig. 4b. (all R^2^ > 0.71, all *p* < 0.01), but none of the intensity groups differed from each other (all *p* > 0.05).

**Table 2:** K^trans^, k_ep_, relative peak intensity, outflow percentage of the differential diagnoses are given with median value, or the precise value(s) if *n* < 3. The total number of different TIC types and T1-weighted intensity types are indicated as well. TIC = Time intensity curve, PEHCR = Peripheral exudative hemorrhagic chorioretinopathy. *quantitative parameters of haemangiomas are exploratory due to poor PKM fit. **Two melanocytomas and two PEHCR had minimal enhancement, resulting in a K^trans^ and v_e_ of approximately 0 and a relative peak intensity of approximately 1.0, therefore k_ep_ and outflow percentage was not calculated for these lesion

Diagnosis	*N*	K^trans^ [min^− 1^]	k_ep_ [min^− 1^]	Relative peak intensity [-]	Outflow percentage [%]	TIC type Wash-out / Plateau / Progressive / Minimal enhancement	T1-weighted intensity Hyper / Iso / Hypo
Haemangioma*	3	6.35*	3.80*	5.86	-34	3 / 0 / 0 / 0	0 / 2 / 1
Multiple myeloma	1	1.11	3.18	3.37	-4	1 / 0 / 0 / 0	0 / 0 / 1
Uveal melanoma	11	0.68	2.45	1.67	-33	10 / 1 / 0 / 0	6 / 3 / 2
Melanocytoma**	3	0.00	1.82**	1.00	-13**	0 / 1 / 0 / 2	3 / 0 / 0
Lymphoma	3	0.35	1.65	1.91	-17	2 / 1 / 0 / 0	0 / 0 / 3
Adenoma	1	0.73	1.63	2.45	-14	1 / 0 / 0 / 0	0 / 1 / 0
Leiomyoma	1	0.15	1.41	1.53	-2.8	0 / 1 / 0 / 0	0 / 1 / 0
Naevus	4	0.20	1.23	1.72	1.8	2 / 0 / 2 / 0	1 / 0 / 3
Metastases	4	0.28	1.06	1.95	5.8	1 / 2 / 1 / 0	0 / 0 / 4
PEHCR**	4	0.07	0.95**	1.20	24**	0 / 2 / 0 / 2	2 / 1 / 1
Medullo-epithelioma	1	0.17	0.67	1.58	57	0 / 0 / 1 / 0	0 / 0 / 1
Schwannoma	2	0.12, 0.14	0.26, 0.36	1.66, 1.72	87, 93	0 / 0 / 2 / 0	0 / 1 / 1
Total	38	0.35 (0.00–6.90)	1.67** (0.26–5.29)	1.66 (1.00–7.96)	-16** (-51 to 93)	19 / 7 / 8 / 4	12 / 9 / 17

**Fig. 5:**
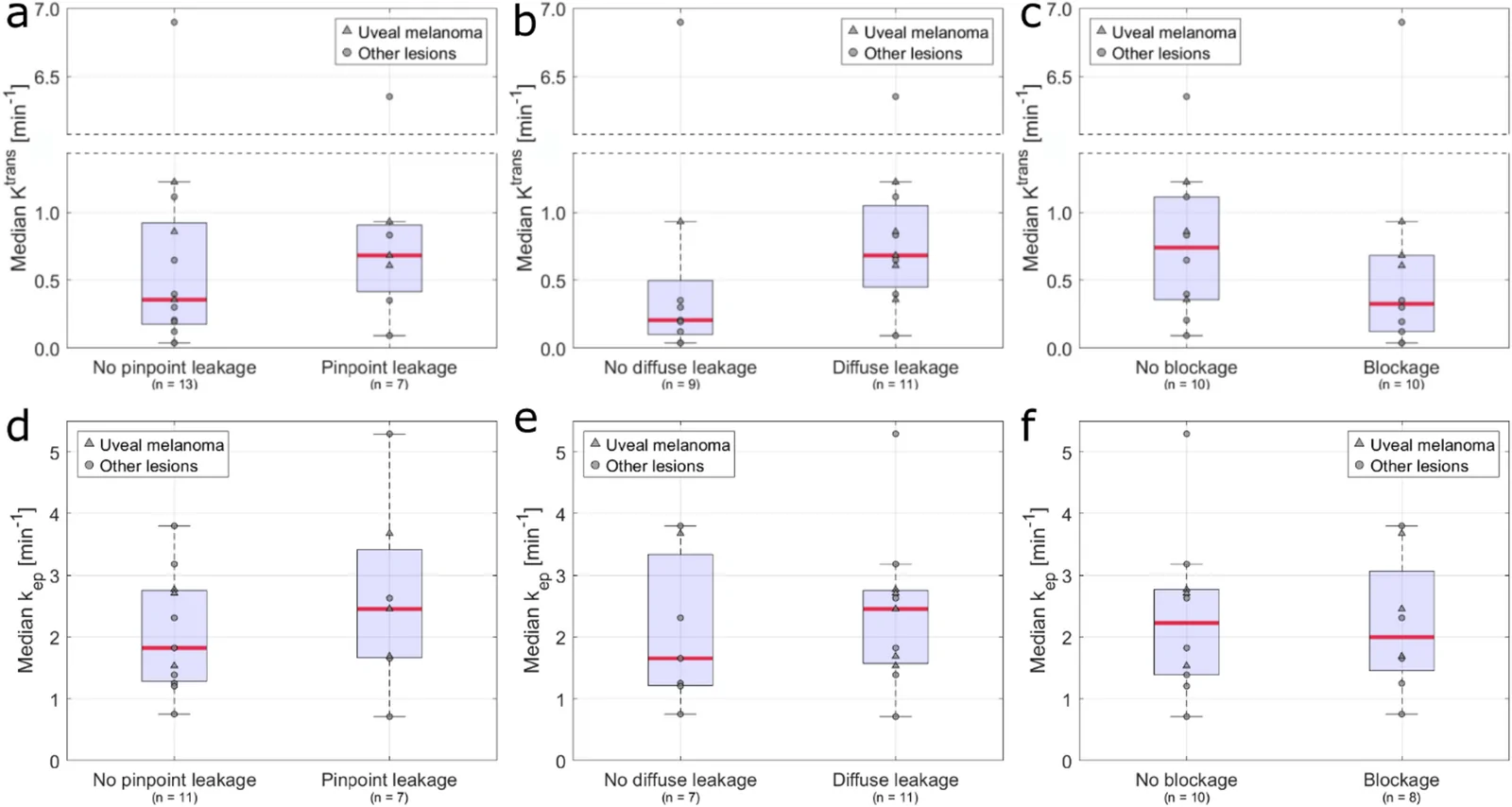
Comparison of median K^trans^ (**a**,** b**,** c**) and median k_ep_ (**d**,** e**,** f**) per lesion of exemplary cases with features visible on FAG images of subgroup that relate to vascularization of the lesions. FAG features include pinpoint leakages (**a**,** d**), diffuse leakages (**b**,** e**) and blockage of fluorescein signal (**c**,** f**). The number (n) per subgroup are indicated in the figure. K^trans^ was assessed in all lesions with FAG images (*n* = 20), while k_ep_ was available for a smaller subset (*n* = 18) due to the exclusion of two lesions with minimal enhancement

The FAG images of 20 lesions were evaluated. Pinpoint leakages were observed in 7 lesions, diffuse leakages in 11 lesions, and blockage of fluorescein signal in 10 lesions, with some lesions showing a combination of these features. Lesions with the same diagnosis did not necessarily display identical FAG features, as shown in Supplemental Table 3. For example, two haemangiomas showed pinpoint and diffuse leakage without blockage, whereas another haemangioma did not show leakage but showed blockage of fluorescein signal instead, possibly due to the chronicity of the lesion. Overall, K^trans^ appeared higher in lesions with pinpoint and diffuse leakages (Fig. 5a and b), and lower for lesions showing blockage of fluorescein signal (Fig. 5c). This is also the case for k_ep_, (Fig. 5d, e and f), although differences in k_ep_ between lesions with or without leakage or blockage were less pronounced.

## Discussion

Several imaging techniques are clinically available to evaluate the perfusion and vascularization of intraocular lesions. Optical FAG imaging is used to assess vascular leakage and blockage at the lesion’s surface, while MRI-based PWI allows for both semi-quantitative and quantitative assessment of perfusion throughout the entire lesion volume. Although semi-quantitative parameters are commonly used in clinical practice due to their simplicity, quantitative parameters derived from PKM often offer a more direct measure of tissue perfusion.

In this study, both quantitative and semi-quantitative PWI analyses were applied across a broad range of intraocular lesions and compared to FAG characteristics. To our knowledge, this is the first study to report PWI parameters for intraocular manifestations of multiple myeloma, melanocytomas, lymphomas, leiomyomas, naevi, metastases, PEHCR, and schwannomas. These results expand the limited perfusion data for these rare entities and contribute to a broader characterization of their imaging profiles, albeit in small case numbers.

Varying perfusion characteristics were observed across lesion groups and were largely consistent with existing literature. Overall, quantitative and semi-quantitative PWI parameters of UM corresponded well with previously reported values [13, 33]. Several UM included in this study were previously described by Klaassen et al. [20] and similar perfusion characteristics were observed. The one intraocular medulloepithelioma demonstrated K^trans^ and v_e_ values within the range reported by Galluzzi et al. [34], as well as a progressive TIC pattern consistent with earlier observations. Choroidal haemangiomas showed very high K^trans^ and k_ep_ values, reflecting their highly vascularized nature [35]. In addition, choroidal haemangiomas presented wash-out TICs and high relative signal intensity compared to UM and naevi, as previously described [36]. Su et al. [37] exclusively reported plateau TIC patterns for retinal pigment epithelium adenomas, contrary, the benign adenoma in our study was a ciliary body adenoma presenting with a wash-out TIC.

Malignant lesions such as UM and multiple myeloma generally demonstrated elevated K^trans^ and k_ep_ values, whereas benign lesions including schwannomas and PEHCR showed lower perfusion values. This pattern aligns with findings in orbital lesions [15, 17], although it was not consistent across all entities. Notably, some intraocular metastases exhibited lower K^trans^ and k_ep_ values despite their malignant nature. These lesions appeared more heterogeneous on contrast-enhanced T1-weighted imaging. However, this heterogeneity was not fully reflected in full volume voxel-based perfusion analysis, as median values over the entire volume were used. This also explains why, in clinical practice, regions of interest are typically drawn in areas showing the strongest enhancement. As for UM, these regions are proposed to represent the more aggressive parts of the tumour [32, 38] and taking the full volume as region of interest dilutes the enhancement.

A key finding in this study is the strong correspondence between semi-quantitative and quantitative PWI parameters. Consistent with previous work [20, 39], higher K^trans^ values were associated with higher relative peak intensity. However, this relationship varied depending on lesion pigmentation and thus on T1 signal intensity. Hypo- and isointense lesions on T1-weighted imaging showed a similar relationship between K^trans^ and relative peak intensity, suggesting that relative peak intensity can serve as a reliable proxy for K^trans^ in these cases. In contrast, hyperintense (typically pigmented) lesions demonstrated a steeper relationship, with similar relative peak intensity values corresponding to higher K^trans^. This confirms and extends earlier observations in UM [20] and indicates that pigmentation affects the interpretation of semi-quantitative perfusion metrics across intraocular lesions more broadly. Contrary, the relationship between k_ep_ and outflow percentage was consistent across T1 signal intensity groups. Taken together, these findings suggest that semi-quantitative PWI analysis may show comparable trends for the clinical assessment of lesion vascularity and differential diagnosis, provided that reference values for relative peak intensity or K^trans^ are stratified according to lesion pigmentation.

When comparing FAG to quantitative PWI, leakage patterns observed on FAG were associated with higher quantitative PWI parameters, whereas blockage of fluorescein signal corresponded to lower perfusion values. This relationship aligns with the expected relationship between FAG features and perfusion, as pinpoint and diffuse leakages on FAG reflect increased vascular permeability, while blockage indicates reduced perfusion or masking by pigment or hemorrhage. Nonetheless, differences in quantitative PWI parameters between FAG feature groups were relatively small. This likely reflects that FAG only visualizes surface vascularization, whereas PWI assesses perfusion throughout the entire lesion’s volume, capturing deeper and more heterogeneous perfusion patterns. Moreover, PWI can be performed regardless of lesion location within the eye, whereas FAG may be limited by lesion location and media opacity.

It is important to acknowledge certain limitations of the used methods. First, there is selection bias, as only intraocular lesions with an available MRI scan could be included. In addition, partly due to the retrospective nature of the study, only a subset of the patients had FAG images available. Second, histopathological confirmation of diagnosis was available for a subset of patients. This reflects clinical practice in ocular oncology [30] where diagnostic biopsies are generally avoided due to the associated risks of complications not performed for diagnostic purposes [22]. The clinical diagnosis of the included patients was, however, substantiated by follow-up information on the subsequent clinical course (Supplementary Table 1). The reluctance to perform biopsies also limits the validation between T1-based signal intensity and pigmentation for naevi, melanocytomas and PEHCR, as these benign lesions seldom get enucleated. The primary quantitative limitation is that, similar to earlier studies [13, 20], a population-based AIF was used instead of an individual AIF, which may introduce variability in quantitative parameter estimation. Also, partial volume effects could occur, particularly in smaller intraocular lesions, where the minimal voxel count may also bias the measured K^trans^ and k_ep_ values. While semi-quantitative PWI parameters of UM have been shown to be reproducible across centers [40], variability due to acquisition and analysis differences should still be considered when interpreting results. Another limitation was the use of the simplified Tofts model, as it assumes negligible plasma volume contribution [14]. This is not the case for haemangiomas, which for a large part consists of blood vessels and blood plasma therefore occupies a substantial portion of lesion’s volume. Consequently, PWI parameters are less accurate in these cases, as was also observed by a lower agreement between the model’s fitted perfusion curve and the actual measured data. The extended Tofts model [14] would offer a better fit by incorporating plasma volume, however, this approach adds to the complexity of the fit and decreases its stability. However, applying the extended Tofts model to all lesions likely does not provide additional clinical value, as the parameters of the haemangiomas already significantly differ from all other lesions. Additionally, no interobserver reliability was reported on FAG assessment. Lastly, due to the exploratory nature of this study, the number of lesions per diagnosis was small, and thus no statistical comparison, evaluation of diagnostic power, external validation or correction for multiple testing could be performed. Nevertheless, these results represent an important step toward better characterization of these rare lesions.

## Conclusion

In this exploratory study, we evaluated the perfusion and vascularization characteristics of intraocular lesions, using both PWI and FAG. Higher perfusion on PWI was more frequently associated with leakage patterns on FAG, whereas lower PWI perfusion values corresponded to fluorescein blockage. In addition, we provided quantitative PWI data of a broad range of intraocular lesions. Overall, malignant lesions showed higher perfusion parameters compared to benign lesions, but some exceptions were present, like the benign haemangiomas. Semi-quantitative PWI parameters may provide similar information on lesion vascularity when stratified for lesion’s signal intensity on T1. As ophthalmic FAG imaging only visualizes the lesion’s surface, PWI may contribute to differential diagnosis and clinical decision-making.

## Supplementary Information

Below is the link to the electronic supplementary material.

Supplementary Material 1

## Data Availability

Data may be available upon request from the Ophthalmology Science Committee (contact via oog.stafsecretariaat@lumc.nl) for researchers who meet the criteria for access to confidential data.
