# Supplementary material for: Quantitative assessment of perfusion-weighted magnetic resonance imaging for the differential diagnosis of various intraocular lesions
Source: Neuroradiology. 2026 Jun 16;68(8):2491–500. doi: 10.1007/s00234-026-04069-7 (PMC13578002; doi:10.1007/s00234-026-04069-7)
Supplement: Supplementary file 1 [file 234_2026_4069_MOESM1_ESM.docx]

**Quantitative assessment of perfusion-weighted magnetic resonance imaging for the differential diagnosis of various intraocular lesions**

**Journal name:** *Neuroradiology*

Supplementary Table 1: Information on confirmation of diagnosis of the individual patients. PEHCR = peripheral exudative hemorrhagic chorioretinopathy

| **Patient ID** | **Diagnosis** | **Confirmed** | **Note** |
| --- | --- | --- | --- |
| 01 | Adenoma | Histopathology | Biopsy confirmed ciliary body adenoma. |
| 02 | Haemangioma | Clinical diagnosis | Diagnosis based on clinical information of 3 years follow-up. |
| 03 | Haemangioma | Clinical diagnosis | Diagnosis based on clinical information. Photodynamic therapy worked successfully, with regression of the lesion and resorption of subretinal fluid. |
| 04 | Haemangioma | Clinical diagnosis | Diagnosis based on clinical information of 9 years follow-up. |
| 05 | Leiomyoma | Histopathology | Biopsy confirmed leiomyoma. |
| 06 | Lymphoma | Histopathology | Biopsy confirmed lymphoma. |
| 07 | Lymphoma | Histopathology | Biopsy confirmed lymphoma. |
| 08 | Lymphoma | Histopathology | Biopsy confirmed lymphoma. |
| 09 | Medulloepithelioma | Histopathology | Biopsy confirmed medulloepithelioma. |
| 10 | Melanocytoma | Histopathology | Histopathology after enucleation confirmed melanocytoma. |
| 11 | Melanocytoma | Histopathology | Pigmented lesion stable over a period of 6 years, with histopathology initially showing no malignancy in the biopsy. MRI was acquired 10 years after this biopsy, the lesion remained stable the entire time. The lesion remained stable for another 3 years after MRI. |
| 12 | Melanocytoma | Clinical diagnosis | Diagnosis based on clinical information. No signs of progression of the lesion for at least 1 year after MRI. |
| 13 | Metastases; lung cancer | Clinical diagnosis | Biopsy at another hospital confirmed primary lung cancer. Diagnosis of choroidal metastases based on clinical presentation and examination. |
| 14 | Metastases; breast cancer | Clinical diagnosis | Biopsy approx. 20 years ago confirmed primary breast cancer. Diagnosis of metastases based on clinical presentation and examination. |
| 15 | Metastases; non-small cell lung carcinoma | Clinical diagnosis | Biopsy confirmed primary non-small cell lung carcinoma. Diagnosis of metastases based on clinical presentation and examination. |
| 16 | Metastases; neuroendocrine tumor | Clinical diagnosis | Biopsy confirmed neuroendocrine carcinoma. Diagnosis of metastases based on clinical presentation and examination. |
| 17 | Multiple myeloma | Histopathology | Patient was already familiar with multiple myeloma, biopsy of the intraocular lesion confirmed the localization of multiple myeloma. |
| 18 | Naevus | Clinical diagnosis | Diagnosis based on clinical information of 6 year follow-up. No signs of progression of the lesion for at least 3 years after MRI. |
| 19 | Naevus | Clinical diagnosis | Diagnosis based on clinical information of 10 year follow-up. No signs of progression of the lesion for at least 3 years after MRI. |

Supplementary Table 1: Continued

| **Patient ID** | **Diagnosis** | **Confirmed** | **Note** |
| --- | --- | --- | --- |
| 20 | Naevus | Histopathology | Biopsy confirmed naevus. No signs of progression of the lesion for at least 2 years after MRI. |
| 21 | Naevus | Clinical diagnosis | Diagnosis based on clinical presentation and examination of 2.5 years follow-up. No signs of progression of the lesion for at least 1.5 years after MRI. |
| 22 | PEHCR | Clinical diagnosis | Diagnosis based on clinical presentation and examination of 1 year follow-up. |
| 23 | PEHCR | Clinical diagnosis | Diagnosis based on clinical presentation and examination. Perioperative diagnosis during vitrectomy, although no biopsy was taken, and the lesion resolved postoperatively. |
| 24 | PEHCR | Clinical diagnosis | Diagnosis based on clinical presentation and examination of 1 year follow-up. |
| 25 | PEHCR | Histopathology | Diagnosis based on clinical presentation and examination. Histopathology after enucleation confirmed no malignancy. |
| 26 | Schwannoma | Histopathology | Biopsy confirmed schwannoma |
| 27 | Schwannoma | Clinical diagnosis | Patient was already familiar with neurofibromatosis type 2. The clinical course of the disease and lesion remained stable for over 13 years. |
| 28 | Uveal melanoma | Histopathology | Histopathology after enucleation confirmed diagnosis of uveal melanoma. |
| 29 | Uveal melanoma | Histopathology | Histopathology after enucleation confirmed diagnosis of uveal melanoma. |
| 30 | Uveal melanoma | Clinical diagnosis | Diagnosis based on clinical presentation and examination. |
| 31 | Uveal melanoma | Clinical diagnosis | Diagnosis based on clinical presentation and examination. |
| 32 | Uveal melanoma | Histopathology | Histopathology after enucleation confirmed diagnosis of uveal melanoma. |
| 33 | Uveal melanoma | Histopathology | Histopathology after enucleation confirmed diagnosis of uveal melanoma. |
| 34 | Uveal melanoma | Clinical diagnosis | Diagnosis based on clinical presentation and examination. |
| 35 | Uveal melanoma | Clinical diagnosis | Diagnosis based on clinical presentation and examination. |
| 36 | Uveal melanoma | Clinical diagnosis | Diagnosis based on clinical presentation and examination. |
| 37 | Uveal melanoma | Clinical diagnosis | Diagnosis based on clinical presentation and examination. |
| 38 | Uveal melanoma | Histopathology | Histopathology after enucleation confirmed diagnosis of uveal melanoma. |

Supplementary Table 2: Characteristics and results of all thirty–eight included patients and their lesions. Quantitative and semi–quantitative parameters are presented as median (IQR). PEHCR = peripheral exudative hemorrhagic chorioretinopathy

| **Patient ID** | **Age** | **Diagnosis** | **Prominence [mm]** | **Basal diameter [mm]** | **FAG** | **T1 [ms]**  ***Median (IQR)*** | **K^trans^**  **[min^–1^]**  ***Median (IQR)*** | **k_ep_ [min^–1^]**  ***Median (IQR)*** | **Relative peak intensity [–]**  ***Median (IQR)*** | **Outflow percentage [%]**  ***Median (IQR)*** | **TIC type** |
| --- | --- | --- | --- | --- | --- | --- | --- | --- | --- | --- | --- |
| 01 | 39 | Adenoma | 5.4 | 9.6 | No | 1228  (1227–1229) | 0.73  (0.63–0.81) | 1.63  (1.45–1.89) | 2.45  (2.35–2.56) | -14  (-21 – -6) | Wash-out |
| 02 | 68 | Haemangioma | 1.7 | 9.3 | Yes | 1554  (1535–1558) | 0.83  (0.45–1.95) | 2.63  (2.12–3.28) | 2.66  (2.10–3.81) | -22  (-36 – -9) | Wash-out |
| 03 | 58 | Haemangioma | 2.3 | 7.3 | Yes | 2069  (2069–2080) | 6.35  (3.90–11.66) | 5.29  (4.35–6.68) | 7.96  (6.85–9.20) | -36  (-38 – -34) | Wash-out |
| 04 | 20 | Haemangioma | 2.5 | 13.9 | Yes | 1329  (1289–1367) | 6.90  (3.81–11.71) | 3.80  (2.93–5.74) | 5.86  (4.57–6.96) | -34  (-40 – -20) | Wash-out |
| 05 | 24 | Leiomyoma | 6.1 | 12.9 | No | 1627  (1616–1636) | 0.15  (0.12–0.18) | 1.41  (1.22–1.62) | 1.53  (1.47–1.59) | -3  (-10 – 5) | Plateau |
| 06 | 75 | Lymphoma | 7.8 | 12.2 | No | 1231  (1223–1232) | 0.42  (0.36–0.47) | 1.61  (1.38–1.86) | 1.91  (1.84–1.98) | -17  (-24 – -11) | Plateau |
| 07 | 49 | Lymphoma | 1.8 | 21.9 | Yes | 955  (850–1054) | 0.35  (0.22–0.55) | 1.65  (1.43–1.87) | 1.60  (1.42–1.82) | -11  (-21 – 3) | Wash-out |
| 08 | 56 | Lymphoma | 2.2 | 12.8 | Yes | 1271  (1219–1344) | 0.65  (0.56–0.76) | 1.82  (1.68–1.97) | 2.38  (2.23–2.52) | -20  (-27 – -13) | Wash-out |
| 09 | 11 | Medullo–epithelioma | 4.5 | 11.1 | No | 1551  (1540–1580) | 0.17  (0.12–0.22) | 0.67  (0.56–0.75) | 1.58  (1.37–1.76) | 57  (40 – 103) | Progressive |
| 10 | 39 | Melanocytoma | 10.8 | 9.8 | No | 1421  (1406–1449) | 0.00  (0.00–0.00) | - | 1.00  (0.98–1.01) | - | Minimally enhanced |
| 11 | 83 | Melanocytoma | 5.2 | 14.7 | No | 843  (816–877) | 0.28  (0.22–0.40) | 1.82  (1.00–2.53) | 1.41  (1.32–1.55) | -13  (-34 – 29) | Plateau |
| 12 | 30 | Melanocytoma | 2.5 | 3.6 | Yes | 881  (881–881) | 0.00  (0.00–0.04) | - | 1.00  (0.84–1.13) | - | Minimally  enhanced |
| 13 | 57 | Metastases; lung cancer | 5.0 | 15.5 | Yes | 1698  (1688–1731) | 0.09  (0.03–0.16) | 0.74  (0.34–1.07) | 1.39  (1.21–1.70) | 16  (-12 – 45) | Plateau |
| 14 | 81 | Metastases; breast cancer | 3.1 | 17.4 | No | 1354  (1275–1449) | 0.16  (0.04–0.31) | 0.62  (0.20–0.90) | 1.56  (1.13–1.97) | 52  (11 – 108) | Progressive |
| 15 | 56 | Metastases; non-small cell lung carcinoma | 2.4 | 11.5 | No | 1810  (1792–1828) | 0.43  (0.25–0.66) | 1.93  (1.06–2.62) | 2.45  (2.04–2.88) | -22  (-36 – 14) | Wash-out |
| 16 | 61 | Metastases; neuro-endocrine tumor | 3.9 | 17.4 | Yes | 1755  (1728–1773) | 0.40  (0.33–0.48) | 1.39  (1.20–1.60) | 2.34  (2.18–2.56) | -5  (-15 – 7) | Plateau |
| 17 | 71 | Multiple myeloma | 7.5 | 18.5 | Yes | 1669  (1658–1699) | 1.11  (0.93–1.29) | 3.18  (2.65–3.80) | 3.37  (3.14–3.58) | 41  (-47 – -34) | Wash–out |
| 18 | 68 | Naevus | 1.3 | 9.1 | Yes | 1483  (1458–1509) | 0.30  (0.25–0.36) | 1.25  (1.03–1.51) | 2.04  (1.87–2.24) | -5  (-13 – 3) | Plateau |
| 19 | 81 | Naevus | 1.5 | 8.7 | Yes | 1677  (1675–1701) | 0.21  (0.17–0.25) | 1.21  (1.11–1.30) | 1.77  (1.63–1.92) | 10  (-1 – 19) | Plateau |

Supplementary Table 2: Continued

| **Patient ID** | **Age** | **Diagnosis** | **Prominence [mm]** | **Basal diameter [mm]** | **FAG** | **T1 [ms]**  ***Median (IQR)*** | **K^trans^**  **[min^–1^]**  ***Median (IQR)*** | **k_ep_ [min^–1^]**  ***Median (IQR)*** | **Relative peak intensity [–]**  ***Median (IQR)*** | **Outflow percentage [%]**  ***Median (IQR)*** | **TIC type** |
| --- | --- | --- | --- | --- | --- | --- | --- | --- | --- | --- | --- |
| 20 | 68 | Naevus | 1.4 | 5.1 | No | 2265  (2265–2265) | 0.11  (0.09–0.13) | 0.93  (0.79–1.04) | 1.66  (1.51–1.76) | 8  (3 – 17) | Plateau |
| 21 | 59 | Naevus | 0.9 | 7.8 | Yes | 1380  (1380–1380) | 0.19  (0.15–0.25) | 2.31  (1.77–2.69) | 1.49  (1.39–1.61) | -47  (-83 – -27) | Wash-out |
| 22 | 75 | PEHCR | 1.3 | 9.0 | Yes | 1059  (986–1099) | 0.12  (0.09–0.17) | 0.75  (0.56–1.07) | 1.30  (1.23–1.40) | 34  (0 – 67) | Plateau |
| 23 | 77 | PEHCR | 1.6 | 6.6 | No | 1017  (1017–1017) | 0.02  (0.01–0.03) | - | 1.08  (1.05–1.12) | - | Minimally enhanced |
| 24 | 81 | PEHCR | 2.4 | 9.4 | Yes | 1463  (1449–1480) | 0.02  (0.00–0.05) | - | 1.10  (1.04–1.17) | - | Minimally enhanced |
| 25 | 51 | PEHCR | 1.3 | 8.6 | No | 1272  (1266–1275) | 0.20  (0.14–0.24) | 1.14  (0.93–1.58) | 1.54  (1.37–1.68) | 14  (-5 – 22) | Plateau |
| 26 | 33 | Schwannoma | 6.7 | 9.2 | No | 1895  (1889–1918) | 0.12  (0.09–0.15) | 0.26  (0.15–0.35) | 1.66  (1.52–1.78) | 87  (66 – 109) | Progressive |
| 27 | 44 | Schwannoma | 2.6 | 8.2 | No | 1879  (1876–1887) | 0.14  (0.13–0.16) | 0.36  (0.32–0.42) | 1.72  (1.60–1.80) | 93  (70 – 120) | Progressive |
| 28 | 79 | Uveal melanoma | 6.3 | 13.0 | No | 683  (674–700) | 0.69  (0.50–0.88) | 2.71  (1.73–3.80) | 1.65  (1.56–1.74) | -32  (-51 – -13) | Wash-out |
| 29 | 67 | Uveal melanoma | 5.1 | 13.2 | No | 658  (654–669) | 0.23  (0.15–0.44) | 1.33  (1.04–1.55) | 1.30  (1.23–1.49) | -12  (-20 – 6) | Plateau |
| 30 | 71 | Uveal melanoma | 6.7 | 13.6 | Yes | 1483  (1482–1488) | 0.68  (0.56–0.84) | 2.45  (2.12–3.05) | 2.49  (2.32–2.68) | -33  (-40 – -25) | Wash-out |
| 31 | 44 | Uveal melanoma | 9.5 | 18.0 | Yes | 486  (475–495) | 0.36  (0.28–0.44) | 1.53  (1.23–1.93) | 1.26  (1.22–1.31) | -15  (-33 – 5) | Wash-out |
| 32 | 75 | Uveal melanoma | 10.7 | 18.4 | Yes | 1513  (1486–1541) | 1.22  (1.00–1.46) | 2.77  (2.19–3.35) | 3.20  (2.98–3.47) | -35  (-43 – -25) | Wash-out |
| 33 | 59 | Uveal melanoma | 9.9 | 17.8 | Yes | 422  (398–447) | 0.86  (0.63–1.14) | 2.71  (2.24–3.27) | 1.43  (1.30–1.57) | -33  (-46 – -18) | Wash-out |
| 34 | 49 | Uveal melanoma | 6.7 | 15.4 | Yes | 1401  (1396–1407) | 0.93  (0.77–1.06) | 3.68  (3.11–4.29) | 2.65  (2.49–2.83) | -46  (-52 – -38) | Wash-out |
| 35 | 38 | Uveal melanoma | 4.9 | 15.6 | Yes | 1244  (1211–1275) | 0.61  (0.49–0.72) | 1.69  (1.52–1.85) | 2.17  (2.00–2.34) | -19  (-29 – -9) | Wash-out |
| 36 | 66 | Uveal melanoma | 6.3 | 18.1 | No | 1308  (1295–1321) | 0.51  (0.40–0.65) | 2.39  (1.87–2.94) | 1.83  (1.74–1.95) | -28  (-36 – -19) | Wash-out |
| 37 | 74 | Uveal melanoma | 6.6 | 15.9 | No | 651  (647–656) | 0.65  (0.53–0.79) | 3.40  (2.94–3.83) | 1.48  (1.41–1.57) | -51  (-58 – -44) | Wash-out |
| 38 | 82 | Uveal melanoma | 11.4 | 15.7 | No | 596  (575–614) | 0.80  (0.69–0.96) | 2.27  (1.85–2.69) | 1.67  (1.60–1.80) | -39  (-47 – -28) | Wash-out |

Supplementary Fig. 1 Median and IQR of K^trans^ and k_ep_ for all exemplary cases. *quantitative parameters of haemangiomas are exploratory due to poor PKM fit. **Two melanocytomas and two PEHCR had minimal enhancement, resulting in a K^trans^ and v_e_ of (approximately) 0, therefore k_ep_ was not calculated for these lesions and not displayed in the graph

Supplementary Table 3: Patients that had fluorescein angiography available before treatment were evaluated on the presence of pinpoint leakage, diffuse leakage, blockage, and a clear wash-in wash-out pattern, with the latter only being evaluated if an ICG was available

| **Patient ID** | **Diagnosis** | **Pinpoint leakage** | **Diffuse leakage** | **Blockage** | **Wash-in wash-out** |
| --- | --- | --- | --- | --- | --- |
| 02 | Haemangioma | Yes | Yes | No | Yes |
| 03 | Haemangioma | Yes | Yes | No | Yes |
| 04 | Haemangioma | No | No | Yes | No |
| 07 | Lymphoma | Yes | No | Yes | No |
| 08 | Lymphoma | No | Yes | No | - |
| 12 | Melanocytoma | No | No | Yes | - |
| 13 | Metastase; lung cancer | Yes | Yes | No | - |
| 16 | Metastase; neuroendocrine | No | Yes | No | - |
| 17 | Multiple myeloma | no | Yes | No | - |
| 18 | Naevus | No | No | Yes | - |
| 19 | Naevus | No | No | No | - |
| 21 | Naevus | No | No | Yes | - |
| 22 | PEHCR | no | No | Yes | - |
| 24 | PEHCR | No | No | Yes | - |
| 30 | Uveal melanoma | Yes | Yes | Yes | - |
| 31 | Uveal melanoma | No | Yes | No | - |
| 32 | Uveal melanoma | No | Yes | No | - |
| 33 | Uveal melanoma | No | Yes | No | - |
| 34 | Uveal melanoma | yes | No | Yes | - |
| 35 | Uveal melanoma | yes | Yes | Yes | - |
